# Inflammatory Cells in Atherosclerosis

**DOI:** 10.3390/antiox11020233

**Published:** 2022-01-26

**Authors:** Marcelle Mehu, Chandrakala Aluganti Narasimhulu, Dinender K. Singla

**Affiliations:** Division of Metabolic and Cardiovascular Sciences, Burnett School of Biomedical Sciences, College of Medicine, University of Central Florida, Orlando, FL 32816, USA; marcellemehu@Knights.ucf.edu (M.M.); Chandrakala.AlugantiNarasimhulu@ucf.edu (C.A.N.)

**Keywords:** monocytes, macrophages, dendritic cells, neutrophils, lymphocytes

## Abstract

Atherosclerosis is a chronic progressive disease that involves damage to the intima, inflammatory cell recruitment and the accumulation of lipids followed by calcification and plaque rupture. Inflammation is considered a key mediator of many events during the development and progression of the disease. Various types of inflammatory cells are reported to be involved in atherosclerosis. In the present paper, we discuss the involved inflammatory cells, their characteristic and functional significance in the development and progression of atherosclerosis. The detailed understanding of the role of all these cells in disease progression at different stages sheds more light on the subject and provides valuable insights as to where and when therapy should be targeted.

## 1. Introduction

Atherosclerosis is a chronic inflammatory disease [1,2,3], in which the intimal layers of the arteries become thick and laden with fat deposits called atheromatous plaque [4,5,6,7,8]. Monocytes and macrophages are immune cells that were first identified in the development of atherosclerosis; later various types of cells were identified in both animal and human atherosclerotic arteries [9,10]. Evidence suggests that atherosclerosis is initiated by dyslipidemia and the recruitment of monocytes to the intima. Infiltrated monocytes differentiate into macrophages, and initiate inflammation and subsequent foam cell formation by the uptake of modified low-density lipoprotein (LDL) via the scavenger receptors, thereby promoting cholesterol loading [11,12]. Numerous pathological studies suggest that macrophage abundance from an early stage of atherosclerosis to late stages, i.e., from fatty streak lesions to the fibrous plaques, indicates the critical role of these cells in the development and progression of the disease [13,14]. These lipid-laden macrophages play a key role in plaque rupture by producing various pro-inflammatory mediators and reactive oxygen species (ROS) [15]. Furthermore, they produce coagulants that enhance inflammation, platelet adherence, the infiltration of various cell types and furthering disease progression towards thrombosis. Data suggests that disease progression involves smooth muscle cell proliferation, calcium deposits, cells death and plaque rupture [16,17].

It is established that elevated levels of low-density lipoprotein (LDL) and decreased levels of high-density lipoprotein (HDL) are key contributors in the development and progression of cardiovascular diseases (CVDs) [18,19]. LDL promotes the progression of the disease, whereas HDL plays a role in preventing the disease progression as well as increasing the regression. The underlying biochemical reactions of early atherosclerotic lesions from fatty streak formation in the disease progression is still under debate. The oxidation of LDL, ox-LDL is reported to be a major player in atherosclerosis development [20,21,22]. The formation of ox-LDL and its nature in vivo is poorly understood. The oxidation of LDL is a complex process, in which LDL associated proteins and lipids undergo oxidative changes. During this process, polyunsaturated fatty acids (PUFAs), lipids associated with LDL, are oxidized to toxic aldehydes, which act on ε-amino groups of lysine residues of the protein moiety generating Schiff’s bases. This, in turn, changes the inter- and intramolecular crosslinks of ApoB [23,24,25]. This modification of apoprotein B might be the key contributing factor of lipid uptake by macrophages. However, evidence suggests that the oxidized lipids other than oxidized LDL and cell membranes, or cells with a similar type of oxidized lipids, also contribute to lipid accumulation in macrophages. The formation of foamy macrophages contributes to several biological inflammatory processes that play a role in the disease progression. Furthermore, the oxidation of HDL also affects various biological properties, including reverse cholesterol transport (RCT) as well as the process of preventing the oxidation of LDL [26,27]. Additionally, during oxidation, HDL loses its anti-oxidative, anti-inflammatory and anti-thrombotic properties.

**Figure 1:**
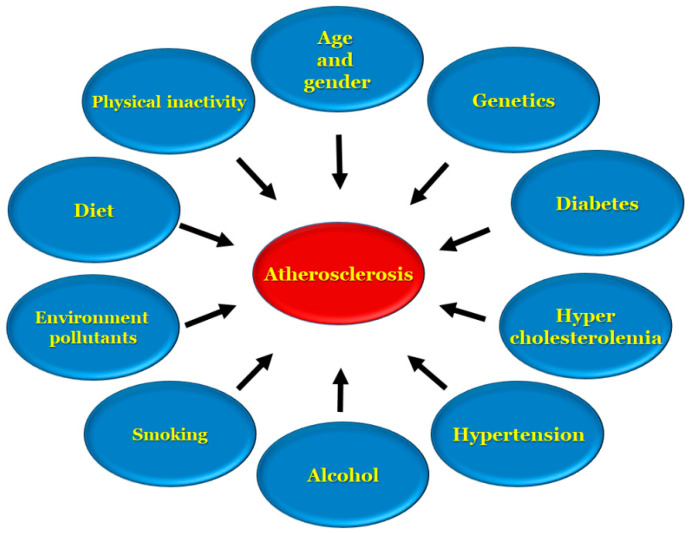
Risk factors associated with atherosclerosis.

Apolipoprotein B is a key factor that remains in the subendothelial layer in which a maladaptive nonrevolving inflammatory process begins, which over the course of time acts as a stimulant for atherosclerosis progression [28,29,30,31,32]. Stroke and heart disease induced by atherosclerosis are two of the leading causes of death in the United States, and are the underlying cause of conditions, such as coronary artery disease, ischemic gangrene, and abnormal aortic aneurysms [3]. There are several risk factors, [4,33,34] involved in the development of atherosclerosis, including age, genetic and environmental factors, high cholesterol, low density lipoprotein presence, high density lipoproteins in low levels, diet, alcohol, smoking, diabetes, hypertension and an inactive lifestyle, as represented in Figure 1.

If there is an incidence of maternal hypercholesterolemia, there is a strong likelihood for the development of atherosclerotic lesions at a young age. When this is coupled with the risk factors mentioned above and other things, such as eating a high fat and fried food diet, these lesions progress at a more rapid rate from early to advanced lesions [35].

Environmental pollutants are another important factor affecting cardiac health globally, where pollutant lead (Pb) is a major risk that causes various cardiovascular diseases. Despite the considerable approaches that have been made to reduce Pb in the environment for decades, the problem persists and affects the health of children and adults. Lead exposure can occur via contaminated food and water, and smoke from the environment, including industrial emissions as well as combustion emissions [36]. Recent studies suggest that the low-level environmental exposure of lead significantly affects cardiac health [36,37,38]. As evidenced by the literature, lead adversely impacts the cardiac system by affecting blood pressure, inflammation and lipid profiles, leading to atherosclerosis. However, this impact varies depending on occupational status [39]. Harari et al. [37] conducted a large cohort study that showed an association between environmental lead exposure and the plaque occurrence in the carotid artery, evidencing the pro-atherogenic role of lead in CVD.

**Figure 2:**
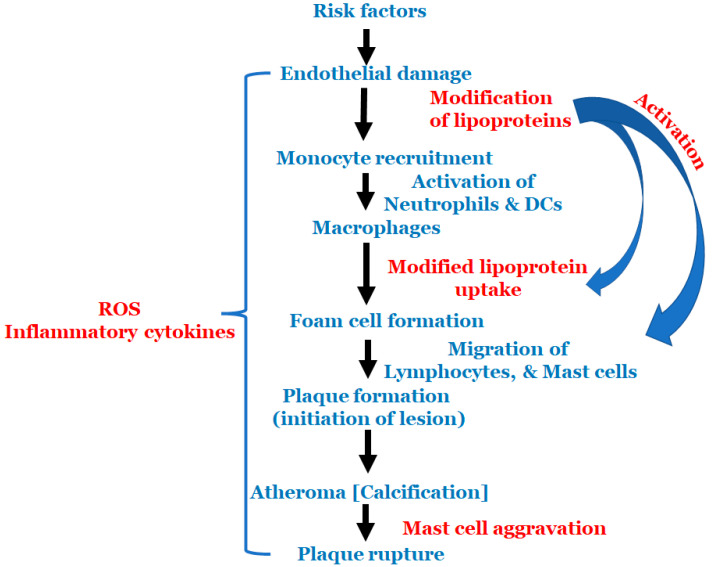
Schematic representation of the events in atherosclerosis.

All the above-mentioned risk factors affect various types of cells that are involved in atherosclerosis. Multiple cell types are active in atherosclerosis, such as monocytes, macrophages, endothelial cells, lymphocytes, smooth muscle cells, neutrophils, dendric cells, foam cells and mast cells. At the start of atherosclerosis, signals are released and monocytes and lymphocytes are recruited to the artery wall, fueling plaque build-up and lesion growth [40]. Low-density lipoprotein (LDL) adheres to the extracellular matrix and accumulates in the intima, the innermost layer of the artery, initiating an inflammatory response involving cells, such as monocytes and lymphocytes [41]. As time progresses, the LDL undergoes modifications, such as oxidation, lipolysis, proteolysis and aggregation, releasing phospholipids and inducing hemodynamic strain [40,41]. Oxidized LDL (ox-LDL) is a major catalyst for atherosclerosis; fat-laden M2 macrophages and monocytes attach to the endothelial cells and form foam cells [40]. T cells are activated by ox-LDL and encounter inflammatory cytokines, such as γ-interferon and lymphotoxin (tumor necrosis factor, TNF–β), which stimulate the macrophages. The macrophages release the monocyte chemoattractant protein-1 (MCP-1), which initiates monocyte migration for attachment and foam cell formation, further progressing the plaque formation. Once present in enough abundance, a proteolytic enzyme capable of degrading the smooth muscle fibrous cap to a thin and weak layer is released and, as a result, it becomes susceptible to rupture [2]. All these processes described above are important to the cascade of cell migration and accumulation that begins with monocyte adhesion and migration into the intima, and ending with a rupture if left untreated. The schema of events is represented in Figure 2.

As atherosclerosis disease has become a major public health issue, a better understanding of atherosclerosis development and progression at the cellular level is needed. This paper reviews the atherosclerotic process at its roots, discussing how the major inflammatory cells work to create the trademark plaque buildup, following the path of cellular activation and their role in the complexity of disease development. With a better understanding of immune cells in the disease development process, and the progression of atherosclerosis, more targeted interventions can be made to prevent, reduce and reverse atherosclerosis lesions.

## 2. Monocytes

Monocytes are white blood cells derived from bone marrow, with the ability to differentiate into macrophages and dendritic cells, and their adhesion to the damaged endothelium is a hallmark of atherosclerotic lesions in its early stages. Ox-LDL stimulates monocytes circulating in the blood to migrate towards the arterial wall [42,43,44,45]. Circulating monocytes are present in three forms based on the levels of cluster of differentiation (CD)14 and CD16 expression (surface cell protein markers): classical monocytes (approx. ~80–95%), intermediate monocyte (approx. ~2–8%) and non-classical monocyte (approx. ~2–11%) [46]. Cellular adhesion molecules on the surface of the endothelial layer, promote the adherence and subsequent migration of monocytes into the arterial walls. The L-selectin on the monocyte cell surface interacts with the P and E selectin of the activated endothelium facilitated by monocyte expressed integrins β1 and β2 for firm adhesion [42]. Both mice and human monocyte markers of different subsets are represented in Table 1.

Monocyte migration into the arterial wall is considered as one of the early events in atherogenesis, which continues in every stage of the disease progression [48]. Monocytes are the precursors of macrophages, and dendritic cells (DCs); migrate into the areas of “injury” by chemotactic stimuli, such as monocyte chemoattractant protein 1 and 3 (MCP-1 and -3). There are 3 major roles that monocytes play in atherosclerosis progression. First, they play a role in the long-term process of the initiation and formation of atherosclerotic plaque by traveling to the site of injury where adhesion occurs, while simultaneously in the subendothelial space they differentiate into macrophages that ingest oxidized LDL via scavenger cells to create foam cells. Second, they play a role in the acute inflammatory phase that follows the destabilization and rupture of atherosclerotic plaque and acute thrombus formation. This causes the thinning of the fibrous cap due to enzymes, such as matrix metalloproteinases (MMPs) and monocyte platelet aggregates that contribute to coagulation cascade in thrombus propagation [49]. Lastly, they play a role in the healing process, specifically in the myocardial tissue during the hypoxic phase, where it promotes various beneficial or detrimental inflammatory processes. Reactive oxygen species (ROSs) are leaked, which stimulate inflammatory cell infiltration [47]. Each of these roles can be attributed to a classification of monocytes, which in turn will become types of macrophages during atherosclerotic progression (Table 1).

Monocyte adherence, their differentiation into macrophages/dendritic cells, polarization into anti- and pro-inflammatory macrophage subsets and the development of scavenger receptors, are implicated in the pathophysiology of atherosclerosis [50,51]. Most monocytes that differentiate will become macrophages; a smaller portion, however, will become monocyte-derived dendritic cells. The differentiation process depends on the cues from the tissue in the micro-environment, such as growth factors and cytokines, particularly the macrophage-colony stimulating factor (M-CSF) and the granulocyte macrophage colony stimulating factor (GM-CSF). These cells are in areas that are not neovascularized compared to the blood-derived dendritic cells that are only in the neovascularized areas [42]. It was shown that the level of phosphorylated p38 MAP kinase can influence the differentiation, causing it to be reduced when present in high levels [52] The exact mechanism that leads to the differentiation is not known, but it is proposed that the non-classical monocytes expressing the chemokine receptors CCR5 and CXCR1 (C-X-C motif chemokine receptor 1), once having passed through the vessel wall, are the best candidate to become monocyte-derived dendritic cells [46]. Monocyte mobilization to atherosclerotic plaque is mediated by several chemokines, among them CCR2, CCR5 and CX3CR1, which majorly contribute to disease progression [53]. The CCL2:CCR2 axis is best described, since CCL2 plays a major role in the recruitment of classical monocytes in plaque [54]. Hence, targeting this axis can help to prevent monocyte recruitment and disease progression. The role of non-classical monocytes in maintaining the vascular homeostasis via patrolling behavior is remarkable [55,56].

A major advancement in the field was the identification of monocyte patrolling, visualized using the intravital multiphoton microscopy [56,57]. Recent studies demonstrate that non-classical monocytes play a key role in maintaining endothelial strength in NR4A1 knockout mice [58,59]. Furthermore, a study by Marcovecchio et al. suggests that kindlin-3 can control the interaction of monocytes with endothelium, which might be a good model to distinguish the specific function of the interaction between non-classical monocytes and endothelium to other potential functions of monocytes [60]. Although, several advances were made in understanding the development, phenotypic nature and functional role of monocytes using RNA sequencing [61], few questions still remain unknown, such as monocytes kinetics and the contribution at different stages of disease progression with age. Multi-omics, as well as epigenetic approaches, might provide a better understanding of the heterogeneity of monocytes and their interactions in atherosclerotic plaques at various stages.

## 3. Macrophages

Macrophages present antigens to cells that initiate inflammation by releasing cytokines; these cells specialize in the detection of dying cells, phagocytosis and the destruction of bacteria [62,63,64]. Their role as an immune cell is crucial; involved in all these processes, they allow for the efficient maintenance of efferocytosis, allowing them to aid in resolving inflammation and prevent the formation of the necrotic core in plaques [65]. This is further evident in the plasticity they possess to turn on their involvement in the inflammatory process when needed [28].

As with many of the cells involved in atherosclerotic lesion formation, macrophages are divided into multiple subsets, M1 and M2 being the two predominant subsets (Table 2). Mox macrophages are known as atherosclerosis-associated macrophages that act as antioxidants, and M4 macrophages reduce phagocytosis and are proinflammatory, playing a minor role [62,64]. In addition, recent studies demonstrated adipose-associated tissue macrophages (ATMs) and tumor-associated macrophages in relation to particular diseases. M1 macrophages are classically activated, typically by the induction of TH1 cytokines, such as the tumor necrosis factor and IFN-γ, participating in the removal of pathogens, activating the nicotinamide adenine dinucleotide phosphate (NADPH) oxidase system and subsequently producing the reactive oxygen species (ROSs). M2 macrophages are alternativity activated and act to counter the effects of M1 macrophages, as well as possessing pro-angiogenic and pro-fibrotic properties. Three subclasses have been identified, M2a, M2b, and M2c induced, respectively, by TH1 cytokines IL-4, IL-13 and IL-10. These M2 macrophages scavenge debris and apoptotic cells, and promote tissue repair and healing [65]. As apoptosis occurs, lipids release their inner contents, leading to the formation of a pro-thrombotic necrotic core [66,67]. The amount and presence of macrophages in the plaque vary; the lesion shoulder is one of the most unstable areas in the plaque, composed primarily of M1, while the necrotic core in comparison is composed of both M1 and M2 macrophages, and the adventitia is mainly composed of M2 macrophages. Furthermore, symptomatic patients exhibit the majority of M1, while asymptomatic patients exhibit M2 as the majority. These macrophages change the phenotype expression over time, depending on the location and microenvironment, all of which modulate the plaque progression and composition [65].

As the lesions progress, the role of the macrophages changes. In the early stages, lipoproteins are accumulated, and the macrophages transform into foam cells or become apoptotic. In the advanced stages, efferocytosis loses its ability to effectively handle the apoptotic macrophages, thus it occurs uncontrollably, expanding the necrotic core. This expansion coupled with the secretion of proteases promotes the rupturing of the plaque. Macrophages can take on a pro-resolving role, where they act to limit macrophage accumulation in the lesions. The progression of atherosclerosis is to a certain degree based on the balance of pro-inflammatory and pro-resolving mediators [28]. The scavenger receptors produced by the macrophages can recognize and process the LDL by binding the ox-LDL, degrading it into its lipoprotein components, in which the cholesteryl esters are hydrolyzed releasing cholesterol and fatty acids. The free cholesteryl is re-esterified by acyl CoA cholesterol ester transferase (ACAT) to cholesteryl fatty acid esters that are the foam portion of foam cells [66].

Recent studies utilize the advances in technology of cytometry by time of flight (CyTOF) and single-cell RNA sequencing, to define macrophage subsets that reside in the atherosclerotic plaques of both mice and humans [68,69,70,71,72]. Based on these studies, the macrophage subsets in atherosclerotic plaques are mainly categorized into three types: resident-like, inflammatory and foamy TREM2hi macrophages [73]. Furthermore, these studies provide a better understanding of macrophage subset-specific markers and their functions in atherosclerotic plaques, which can guide us in the development of new therapeutic strategies to prevent atherosclerotic plaque progression. The study of Gong et al. [74] suggests that M1 macrophages are predominant in vulnerable plaques, and the activation of STAT3/6 switches the M1 phenotype to M2 and leads to plaque regression. This study suggests that the therapeutic strategies of switching macrophage phenotypes can help in plaque regression. Recent clinical trials demonstrated that cholesterol lowering drugs, such as statins and proprotein convertase subtillisin/kexin type 9 (PCSK9) inhibitors, significantly promote plaque regression and retard plaque progression [75]. This might not only be due to the efficacy of these drugs in lipid lowering, but also these drugs might be playing a role in switching the macrophage phenotype and causing plaque regression, which requires further research with advanced technologies under treated conditions.

## 4. Foam Cells

Foam cells present in the lesion are mainly a product of M2 macrophage differentiation forming foam cell clusters in the early stages. Initially functioning as a mechanism to remove the cytotoxic and pro-inflammatory ox-LDL in the intima, the macrophages are unable to process the increased influx of ox-LDL and accumulation of cholesteryl ester [42,76,77]. Ox-LDL accumulation in the endothelial barrier is facilitated by protein kinase C activation, which increases the permeability of the endothelial layer. Once in the barrier, the macrophages undergo phagocytosis and pinocytosis, becoming foam cells [76]. As the foam cells accumulate in the plaque shoulders and necrotic core, metalloproteinases are released that facilitate plaque destruction, rendering them increasingly unstable [42].These macrophages also increase their expression of lectin-like oxidized low-density lipoprotein (LOX-1) and ATP-binding cassette transporters, such as ABCA1 and ABCG1, which are involved in the decrease in reverse cholesterol transport expression and scavenger receptors [76]. In the atherosclerotic lesion, two types of foam cells can be found: those differentiated from smooth muscle cells and the others from macrophages derived from blood monocytes. Smooth muscle cells account for most of the foam cells found in the lesions, as they are the main type of cell found in intimal thickening. The presence of the two types of foam cells at the different stages of atherosclerosis changes between humans and mice. For example, macrophage foam cells in mice are seen in the initial stage, while in humans they do not become apparent until a major deposit of extracellular lipids has been made in the intima [78]. The calcification of the arteries also occurs as the foam cells infiltrate into the intima and media, increasing the vascular smooth muscle cells and extracellular matrix mass [76]. This is the biomineralization process by which insoluble calcium, primarily in the form of calcium salts, is deposited in the plaques. It is proposed that calcification’s role in atherosclerosis may be to act as a stabilizing force for the plaque, mechanically and biochemically, by adding increased rigidity to the area. Proteoglycans have been shown to possibly play a role in calcification. They act as a calcium-concentrating agent due to their ability to bind calcium [79].

The recent studies of Wang et al. [80] and Kim et al. [69] reported that the majority of foam cells in both human and mouse atherosclerotic lesions are from SMCs identified by single cell RNA sequencing (scRNAseq), whereas Winkles et al. [68] showed that 65% of foam cells in atherosclerotic lesions are of a monocyte/macrophage origin observed using scRNAseq, which are conflicting and requires further research. These studies suggest the requirement of advanced techniques, such as the multi-omics approach, which can help to define the origin of various types of foam cells present in lesion initiation to progression, and to understand the underlying regulatory mechanisms involved in foam cell formation, which will shed light on developing new therapeutic approaches to prevent the late-stage complications of diseases, such as thrombosis. Furthermore, Wang et al. [81] studies suggested that natural products, namely flavonoids, terpenoids, phenolic compounds, phenylpropanoids, alkaloids, steroids, fatty acids, amino acids, carbohydrates and several others, are better therapeutic approaches to target foam cell formation.

## 5. Lymphocytes

A type of white blood cell, lymphocytes, are present in the form of B cells and T cells (Table 3). The innate and adaptive immune responses, both humoral and cellular, showed the involvement of B cells [82,83,84,85,86]. Composed of several subsets, B1 cells are atheroprotective and B2 cells are proatherogenic, comprising the larger portion of the total B-cell population [87]. The peritoneal and pleural cavities house the B1 cells, and, as part of their atheroprotective nature, they play a role in innate immune response and the secretion of large quantities of natural IgM antibodies. B2 cells are housed in the spleen and lymph nodes as follicular B cells (FOBs) or marginal zone B cells (MZBs). The atheroprotective nature of B cells was confirmed by experiments, in which the damage to the B cells either completely or partially lead to exacerbated atherosclerosis. FOB cells are proposed to exhibit different properties, protective or enhancing, based on the state of inflammation and the local environment. When anti-CD20 antibody treatment was given to hypercholesterolemic ApoE-/- and Ldlr-/- mice, their showed decreased lesion formation, while transforming splenic B2 cells into lymphocyte-deficient Rag2-/-γ-chain-/-ApoE-/- recipients, showed enhanced atherosclerosis. MZBs have a low level of activation, making them active in the early immune response in greater quantities, and they can uptake ox-LDL. A notable ability of B cells is that they undergo hyper somatic mutations to become antibody-producing cells. The antibody nature of the cells allows them to serve as receptors for antigens. T-cell activation is directly regulated by the presentation of antigens, co-stimulation and cytokine production [82].

Undoubtedly, B cells are impactful to the progression of atherosclerosis; however, T cells are the type of lymphocytes that play an even larger role. T cells are present early in the disease, with the expression of CD4+ T cells or T helper cells (Th) that produce IFN-γ and TNF-α, which increase over time [87,89]. In studies with CD4+ T cell-deficient Apoe^−/−^ mice, a reduction in the lesions in the aortic root was observed. Th cells are heterogeneous in nature, including both pro- and anti-inflammatory cells [90]. The T cells are divided into CD4+ Th1 and CD4+ Th2 cells. Th1 cells are induced by the cytokines interleukin IL-12 and IL-18, which increase atherosclerosis. Th2 protects the cells; however, if IL-5 and IL-3 are deleted, the disease continues to progress as Th1, which is the most abundant type to be found at the lesion sites. It promotes the growth of the lesion, as well as its destabilization by altering the endothelial function, recruiting inflammatory cells within the lesion and interfering with cholesterol export from the cells within the lesions [87]. The expression of the Th1-specific transcription factor coupled with signal transducer and activator of transcription 4 (STAT4), leads to the differentiation of Th1 cells and are pro-atherogenic. When IFN-γ and TNF-α are absent from the hypercholesterolemic mice, there is reduced atherosclerosis, providing evidence of the pro-atherogenic nature of Th1 [90]. Th2 inhibits Th1 differentiation and subsequent IFN-γ secretion, suggesting that it has a protective role. It is also found that it can modulate the development of coronary artery disease [87]. Th2 interacts with B cells, differentiation is initiated by IL-4, which inhibits IFN-γ production and in turn induces Th1 differentiation. CD4+ regulatory T cells (Tregs) are another heterogeneous group of cells that suppress the pathogenic immune response. Present in low numbers in atherosclerotic plaques, Tregs serve a protective role [90]. Yet another T-cell subset, Th17 has also been observed to play a role; however, the exact role is still being debated. The production of 1L-17A from Th17 has been shown to be active in plaque inflammation and instability [87]. Although the role of Th17 is still unknown, it is suggested that it targets collagen V, thus exacerbating atherosclerosis as well as interacting with Tregs cells to create a balance that, if disrupted, also progresses atherosclerosis. [90]. Furthermore, Butcher et al. [92] reported that Western diet fed *Il17a*^−/−^*Apoe*^−/−^ mice and *Il17ra*^−/−^*Apoe*^−/−^ mice had smaller plaques in the aortic arch and aortic roots, compared to control *Apoe*^−/−^ mice, whereas the abdominal aorta showed a similar plaque burden. In contrast, Taleb et al. [93] reported that the administration of IL-17A showed a reduced plaque burden in LDLR KO mice. Evidence suggests the involvement of T-cell subsets and their functions in atherosclerotic plaques, specifically in experimental models. Nevertheless, there is much more to know since it is challenging to translate their mouse model disease condition to humans [94]. Hence, targeting T cells and secretory factors can be a new preventive strategy and therapeutic approach to treat atherosclerosis.

## 6. Neutrophils

Neutrophils are highly efficient effector cells that are part of the first line of defense in the innate immune response when the body is undergoing an acute inflammatory process. They possess the ability to fight many pathogens and signal molecules for use in secondary immunity. The early stages of atherosclerosis are characterized by the dysfunction of the endothelial process and the adhesion of molecules as they are upregulated, with neutrophils traveling from the bloodstream to the vascular wall. As the neutrophils roll along the surface, they start to form slings, which are cell-autonomous adhesive structures that extend at the front of the rolling neutrophil as anchor points, and, along with tethers, they enable the rolling. Chemokine CCL5 binds to CCR1 and CCR5 that, along with CXCR2 and CCR2, facilitate firm adhesion and subsequent neutrophil emigration [95,96]. Neutrophils possess four different granule subsets that are used for immune regulation via the release of proteins: primary, secondary, tertiary and secretory vesicles. Primary granules in young cells are the main location for the storage of toxic mediators and secondary granules in mature cells containing lactoferrin and matrix metalloprotease 9 [97]. Once the neutrophils enter the extravascular tissue, the primary and secondary granules are released. As the neutrophil–endothelial interactions begin, the secretory vesicles are mobilized, and during neutrophil transendothelial migration, there is the release of tertiary granules [98]. The mice- and human-specific markers of neutrophils, as well as their characteristic functions, are represented in Table 4.

Neutrophils, over time, undergo apoptosis, which reduces the inflammatory process. However, if the macrophages are no longer able to phagocytize, then the neutrophils become necrotic, adding to the inflammation. At this point, the cells can undergo neutrophil extracellular trap activation and release (NETosis), a defense mechanism in which there is a release of the cytosolic granule proteins bound to nuclear material that will fight the foreign pathogens. Though more research is needed, it has been shown that NETosis potentiates atherosclerosis by macrophage priming and cytokine release to activate Th17 cells, as well as forming a scaffold [100]. The ratio of neutrophil to lymphocyte, is a marker of inflammation (increased neutrophils and decreased lymphocytes) that has gained attention in recent years. Using this ratio, it is possible to predict the occurrence, progression and apoptosis in atherosclerosis while it is still stable. This is an important marker because the neutrophils promote the progression of atherosclerosis, while lymphocytes, particularly B lymphocytes, reduce the progression [99].

Neutrophils play a significant role in the destabilization of atherosclerotic plaque by NETs-induced SMC lysis or death by histone H4 [101]. Furthermore, this study suggests that the neutralization of histone H4 might be helpful in preventing SMC cell death as well as the destabilization of atherosclerotic plaques. The recent study of Gomez et al. reported that the microvesicles of neutrophils play a significant role in promoting inflammation and atherosclerosis. These microvesicles carry miR-155, which can enhance NFKB activation and inflammatory gene expression at disease-prone sites [102]. The studies of Geng et al. [103] demonstrated how endotoxemia affects neutrophils to a non-resolving inflammatory state, with the increased inflammatory mediators of leukotriene B4 (LTB4), matrix metalloproteinase (MMP9) and dectin-1, which are reduced homeostatic mediators. The ROS generated by LPS changed the peroxisome homeostasis and lysosome fusion. Furthermore, this study suggests how the novel reprogramming of neutrophils with 4-phenyl butyrate restored the peroxisome homeostasis and balanced the pro- and anti-inflammatory mediators, which in turn led to the reduced pathogenesis of atherogenesis. These studies raise the concern of the use of therapeutic strategies for the specific components of neutrophils that cause damage without impacting NETosis-mediated host defense.

## 7. Dendritic Cells

The life cycle of dendritic cells begins as progenitor cells in the bone marrow, after which they circulate in the bloodstream until a site for antigen entry is located. In this stage, the dendritic cells are in an immature form in the peripheral tissue, in which they will remain until inflammatory signals are detected. At this junction, the cells transform into mature dendritic cells and migrate into the lymphoid tissue to interact with T cells. Dendritic cells are antigen-presenting cells, which present antigens to the T cells, initiating and maintaining an immune response, or inhibiting the activation. The role that the dendritic cell will play is dependent on the kinds of cytokines that are released and expressed on the cell surface. The activation of the innate immune response receptors, for example, the toll-like receptors, turn the DC into antigen-presenting cells that activate the T effector cells. When the antigens are presented in the absence of the toll-like receptors, immunological tolerance is induced [90].

The main role of dendritic cells is initiating antigen-specific adaptive immune responses, and maintaining tolerance to self-antigens; they are found in the vascular walls and lymphoid tissue. They are classified phenotypically and divided into two groups, classical dendritic cells and plasmacytoid dendritic cells, based on the cell surface markers and functional criteria [104,105,106,107]. Classical dendritic cells are divided into cDC1 and cDC2, identified by the markers CD11b+ andCD8+CD103+ in mice and CD1c/BDCA-1+ and CD141/BDCA-3+ in humans, respectively [104]. The dendritic cells in the non-lymphoid tissue are not as easily categorized as the surface markers, as they are also found to be expressed by some macrophages. For example, macrophage foam cells have been shown to express CD11c and MHC-II, which are dendritic cell surface markers. To circumvent this, identification is performed by testing for multiple known surface markers simultaneously [106]. The classical dendritic cells have the capacity to induce antigen-specific MHCI or MHCII restricted T-cell proliferation, as well as promote tumor necrosis factor-α and interferon-γ production. This leads to the conclusion that dendritic cells may cause T-cell activation and proinflammatory cytokine production. The role of antigen and cytokine activation and release by plasmacytoid dendric cells requires further study [104].

Dendritic cells exist in 3 forms: as precursors in the blood, immature cells in the peripheral tissue and mature cells in the lymphoid tissue [108]. Dendritic cells are actively present in the areas that are prone to be atherosclerotic, such as the lesser curvature of the aorta. Shear-stress signals are suspected to play a role in their recruitment since these areas are areas in which there is high shear-stress blood flow. In the early formation of atherosclerotic lesions, the dendritic cells become foam cells by the uptake of lipoproteins and lipid-laden apoptotic cells, emigrating from the lesion into the draining lymph node to present antigens, as well as activate naïve T cells that are anti-inflammatory. In the advanced atherosclerotic lesions, the emigration of the dendritic cells from the plaque is defective; thus, they start to build up, which can lead to enhanced local inflammation. Alongside this, dendritic cells are poor in efferocytosis, which can lead to the further expansion of the necrotic core. This is due to dendritic cell medicated efferocytosis aiding in the prevention of secondary cellular necrosis, which is a major pro-inflammatory signal, accelerating the lesion and necrotic core formation [106]. This was further explained by a study in which the atherosclerotic segments from hypercholesterolemic ApoE^−/−^ mice were transplanted into wild-type normocholesterolemic recipient mice. The results showed that in normocholesterolemic recipient mice there was lesion regression, whereas in hypercholesterolemic mice, lesion development continued progressively after transplantation. The ability of the dendritic cells to exit the aortic lesion was impaired due to progressing and persistent dyslipidemia in the hypercholesterolemic mice [108]. Different types of dendric cells, cell specific markers and characteristic functions are presented in Table 5.

With the development of modern technologies, such as high-dimensional cytometry by time of flight (CyTOF) and single-cell RNA sequencing (scRNAseq), enables a better understanding of the heterogeneity of DCs [68,71]. These studies identified a third subset that differs from cDC2 with the absence of CD41, and a low expression of CD11b. The recent study of Zhao et al. [109] put together the existing reports and described the myriad of functions of DCs in every step of atherosclerosis development, including lipid uptake, efferocytosis, antigen presentation, immune function and chemokine secretion. Furthermore, this study suggests that the understanding the pathogenic role of the DCs in atherosclerosis will open new therapeutic approaches to prevent CVD.

## 8. Mast Cells

Derived from progenitor cells in the bone marrow, mast cells express specific surface markers, including CD34, CD13 and CD117. CD117 is of importance as it is a receptor for the mast cell growth factor. Mast cells are part of the body’s innate and adaptive immunity, and as they mature they are activated by antigen-sensitized IgE fragments due to the high-affinity IgE receptors they possess. Allergic/inflammatory reactions will trigger them to release their granular load. The material released from the granules consists of pro-inflammatory cytokine, histamine and neuronal proteases [110]. Located primarily in the mucosal and connective tissues, when activated, the cells affect the surrounding vessel wall, provoke matrix degradation and apoptosis [111]. When the cells degranulate in the presence of LDL particles, they result in the conversion of macrophages into foam cells. This is performed by the LDL interacting with heparin in the granules, and these complexes are phagocytosed by cultured macrophages. The macrophages become filled with cytoplasmic lipid droplets of re-esterified LDL-derived cholesterol. These granules also interact with HDL to inhibit their ability to cause high-affinity cholesterol to leave the foam cells [110]. As the lesions form, the mast cell takes residence in the shoulder regions, and the proteases, such as tryptase and chymase that release during degranulation, can cause intra-plaque hemorrhage, vascular leakage and macrophage and endothelial cell apoptosis. The intraplaque hemorrhages can result from the release of angiogenic compounds. These compounds induce the growth of microvessels and lead to the leakiness and ultimate rupture of the neo-vessels [111]. In humans, these proteases are used as a method to characterize the cell. Tryptase is expressed in three forms (α-,β- and γ- tryptase) and chymase in one form. In mice, they are categorized by the location from which they are found: mucosal mast cells located on the mucosal surface and connective tissue mast cells in the skin and perivascular areas of tissue. The two types of mast cells express four types of chymase and two types of tryptase, which are both expressed in the mucosal and connective tissue mast cells. CCR3 is an eotaxin receptor and its inhibition has been shown to limit the accumulation of mast cells, and thus reduces lesion development and recruitment to the plaque. Another factor at play in recruitment is tissue homing by the mast cells found in the perivascular tissue. Alpha4-integrin vascular cell adhesion molecule 1 (VCAM-1) and P-selectin glycoprotein ligand-1 (PSGL-1) selection are needed to bring the mast cell progenitor cells to the area, aiding in the adhesive interaction with cytokine-activated human vascular endothelial cells [110]. Mast cells also express toll-like receptors, such as TLR-8 and TLR-3, which, when activated, release TNF, IFN-γ, IL-6 and IL-8, which then produce chemotactic factors, such as MCP-1. This all leads to the conclusion that mast cell activation is in part an overall activator for the atherosclerotic processes, releasing cytokines that further the accumulation of the other inflammatory cells present [111]. Table 6 represents the mice- and human-specific surface markers along with the specific functions.

The presence of the activated mast cells in atherosclerotic lesions at various stages has been confirmed [112,113,114]; however, there is scant information about the specific exogenous or endogenous antigens in the activation of these cells. Additionally, mast cell causing acute allergic reaction in atherosclerotic plaques is still unknown. However, it has been shown that mast cells harbor IgE receptor-bound IgE antibodies on their surfaces in human myocardium [115]. The recent studies of Kritikou et al. [116] demonstrated the flow cytometry-based characterization of mast cells, and found both IgE-dependent and -independent mast cell activation. The majority of the activated mast cells in plaque samples are IgE-dependent, and suggest that targeting these cells might provide a better therapeutic approach in the prevention of disease. Evidence suggests that the variations in gut microbiota and microbial infections can also increase the risk of CVD [117,118]. The studies of Gupta et al. reported the presence of host defense peptides and the role LPS derived from *Porphyromonas gingivalis* in mast cell activation and degranulation, suggesting the possibility of a microbial role in disease progression [119]. Even though atherosclerosis is a non-infectious disease, these studies pose a question of the possibility of infection accelerated disease progression. It has been also shown that heparin proteoglycans derived from peritoneal mast cells attenuated the thrombus in a rat model, suggesting their anti-atherosclerotic functions [120,121]. Further, these studies signify the essential need to better understand the role of receptors on mast cell subsets, and their role as pro- and anti-atherogenic functions in developing lesions using single-cell isolated technologies, which might be more helpful in the development of therapeutic approaches.

## 9. Conclusions

Inflammation is the human body’s defense for when it is attacked; however, sometimes this defense can lead to a worsening of one’s condition. In atherosclerosis, many of the cells have both inflammatory and pro-inflammatory characteristics, showing how inflammation can work in either direction.

In recent years, the functions of various immune and inflammatory modulators in the formation and development of atherosclerosis have been better evaluated. Additionally, these studies also provide a deeper insight into the underlying pathological mechanisms. Furthermore, the development of diagnostics and prognostics with advanced techniques explores the treatment of atherosclerosis. However, we are still not able to completely prevent or cure the disease; hence, there is much more to learn in-depth about the role of immune cells that are involved in the progression of atherosclerosis. To date, the exact function of immune cells and their function either as pro- or anti-inflammatory agents in disease progression is not yet completely described and is under debate. The oxidative stress, inflammation and metabolic or signaling pathways in various types of cell death have not been established completely under disease conditions, which make this a topic of interest. By understanding the main path that ends with foam cell accumulation and calcification, which is the catalyst for creating an environment prone to rupture, the smaller pathways of inhibition and promotion can be investigated in more depth. With the knowledge of how each inflammatory cell functions individually, the points at which the interactions occur can be better seen as providing specific junctions that can be used for targeted treatment. Some of those points include the neutrophil to lymphocyte ratio, which provide a tangible way to measure lesion development non-invasively, and T cells that have many pathways to activation, all of which further our knowledge of the complex process that is atherosclerosis. In this review, we described all the major immune cells that are independently playing a key role in disease progression. We believe that this review will provide insights with recent updates, which will shed light on the therapeutic approaches of atherosclerosis.

## Figures and Tables

**Table 1:** Monocyte types and function.

Monocyte Type	Human Markers	Mice Markers	Properties and Function	References
Classical (Mon 1)	CD14++ and CD16−	Ly6C++ and CD43+	Highly phagocytic and vital scavenger cells	[46,47]
Intermediate (Mon 2)	CD14++ and CD16+	Ly6Cint and CD43+	Endothelial adherence Antigen presentation Major role in proliferation and stimulation of T cells, inflammatory responses and angiogenesis Pro-inflammatory in nature and secretes inflammatory cytokines	
Non-classical (Mon 3)	CD14+ and CD16++	Ly6C+ and CD43++	Very mobile Monitors endothelial injury Anti-inflammatory in nature	

**Table 2:** Macrophage types and function.

Macrophage Type	Stimulus for Differentiation	Role in Inflammation	Mice Markers	Human Markers	References
M1	Ox-LDL, proinflammatory cytokines, lipopolysaccharides	Proinflammatory	IL-1β, TNF, IL-6, IL-12, IL-23, CXCL9, CXCL10, CXCL11, arginase II	IL-1β, TNF, IL-6, IL-12, IL-23, CXCL9, CXCL10, CXCL11, arginase II	[47,62,64,65]
M2 (a,b,c)	IL-4, IL-10, IL-1β	Anti-inflammatory, resistant to lipid accumulation	Arginase I, resistin-like α, Ym1, Ym2, MMGL, stabilin-1, CD163, IL-10high, IL-12low	MMR, IL-1RA, factor XIIIa, CD200R, CCL18, stabilin-1, CD163, IL-10high, IL-12low	
Mox	Oxidized phospholipids	Antioxidant	HO-1 (heme oxygenase sulfiredoxin-1), TR, NFE2L2 (nuclear factor (erythroid-derived 2)-like 2)	HO-1, sulfiredoxin-1, TR, NFE2L2	
M4	CXCL4 (C-X-C motif chemokine 4)	Proinflammatory, reduces phagocytosis	MMP-7, S100-A8, MMR (macrophage mannose receptor)	MMP-7, S100-A8, MMR	

**Table 3:** Lymphocyte types and function.

Lymphocyte Type	Human Markers	Mice Markers	Characteristics	References
B1 cells	High levels of IgM	High levels of IgM	Active in innate immunity housed in the peritoneal and pleural cavities	
	low levels of IgD, detectable levels of CD5	Low levels of IgD, detectable levels of CD5		[88,89,90,91]
B2, follicular B cells (FOB)	CD10, CD19+ CD20, CD21^mid^, CD22, CD23, CD24^low^	CD1d^low^, CD19^mid^, CD21^mid^, CD22, CD23, CD24^low^, CD38+	Housed in the spleen and lymph nodes Participate in T-cell-dependent immune responses	
B2, marginal zone B cells (MZB)	CD1c, CD19+, CD20, CD21^high^, CD27+, IgM+	CD1d^high^, CD19^mid^, CD21^high^, CD22, CD23, CD35+, CD43−	Housed in the spleen and lymph nodes Active in early immune response and can uptake ox-LDL	
T cells,Th1	CCR1+, CCR5+, CD3+, CD4+, CD8−, CD14−, CD19−, CXCR3+	CCR1+, CCR5+, CD3+, CD4+, CD8−, CD14−, CD19−, CXCR3+	Promote lesion destabilization Alter endothelial function Most abundant type at lesion sites	
T cells,Th2	CCR3+, CCR4+, CCR8+, CD3+ CD4+, CD8−, CD14+, CD19+	CCR3+, CCR4+, CCR8+, CD3+, CD4+, CD8−, CD14+^,^ CD19+	Inhibit TH1 differentiation and promote the survival and proliferation of mast cells	

**Table 4:** Neutrophils and their role in atherosclerosis.

	Mice Markers	Human Makers	Function	References
Neutrophils	Lin-, CD11b, CD45+, Ly-6C	CD11b, CD14low/int, CD15+, CD16, CD32	Central role in innate immunity by destroying foreign particles Ratio of neutrophils to lymphocytes acts as an atherosclerosis progression tool	[91,95,97,98,99]

**Table 5:** Dendritic cells and function.

Types of Dendritic Cells	Mice Markers	Human Marker	Characteristics	References
Classical, cDC1	CD8+CD103+, CD11c, CD45, CD40, CD83, CX3CR1	CD141/BDCA-3+, CD11c, CD14, CCR7	Activate CD8+ T cells	[90,91,104]
Classical, cDC2	CD11b+, CD11c, CD45, CD40, CD83, CX3CR1	CD1c/BDCA-1+, CD11c, CD14, CCR7	Promote Th2/Th17-mediated immune responses	
Plasmacytoid	CD1a-, CD11c^low^, Lin-, IL-3 R alpha, CD123+	Lin-, CD11c+, Ly-6C+	Regulate MHCI/MHCII to activate naïve CD4+ T cells	

**Table 6:** Mast cells and function.

	Mice Markers	Human Makers	Function	References
Mast cells	Lin-, CD34+, CD45+, CD117, CD11c-	Lin-, CD11c-, CD45+, CD33	Housed in mucosal and connective tissues. Part of innate and adaptive immunity.	[91,110,111]

## Data Availability

Data are contained within the article.
